# Development of a Smart Portable Hypoxic Chamber with Accurate Sensing, Control and Visualization of In Vitro Cell Culture for Replication of Cancer Microenvironment

**DOI:** 10.3390/cancers15143645

**Published:** 2023-07-16

**Authors:** Harish Ramachandramoorthy, Tuan Dang, Ankitha Srinivasa, Kytai Truong Nguyen, Phuc Nguyen

**Affiliations:** 1 Department of Bioengineering, University of Texas, Arlington, TX 76019, USA; harish.ramachandramoor@mavs.uta.edu (H.R.);; 2 Joint Bioengineering Program, University of Texas Southwestern Medical Center, Dallas, TX 75390, USA; 3 Department of Computer Science, University of Texas at Arlington, Arlington, TX 76019, USA

**Keywords:** cancer, hypoxia, automated, remote monitoring, visualization, tumor microenvironment, drug resistance

## Abstract

**Simple Summary:**

For many diseases including cancer, translating treatment from laboratory to clinical studies have been an issue. This was due to a lack of adequate in vitro models to replicate in-human disease conditions. Studies have shown severe lack of Oxygen in tumor microenvironment known as hypoxia to be one of such limiting factors. In this research we aimed at designing a device that could create and maintain cultured cells in vitro at specific hypoxic conditions. We confirmed the device was not only capable of creating specified oxygen levels in the cell chambers but also able to maintain the set oxygen levels for an extended period of time. We also showed the relevance of this chamber in creating a cell culture model with biosimilar oxygen level found in tumor microenvironment. This device will thus bridge a gap between in vitro and in vivo analysis of cancer therapeutics.

**Abstract:**

Clinical resistance towards treatment is a major concern in cancer therapy. This is due to in vitro studies lacking essential microenvironmental aspects. Tumor-hypoxia is an important pathophysiological phenomenon in numerous malignant tumors. Various studies have shown the importance of a hypoxic microenvironment (HME) in cancer drug resistance and its effects on cellular signaling and metabolism pathways. Most drugs fail in transition from a laboratory to clinical trials because of the variability in the testing microenvironment conditions. It is, thus, very crucial that research work needs to replicate these conditions in vitro to test the drugs and/or drug carriers for cancer therapy. Previous works have used a portable hypoxia chamber to reduce the cell microenvironment to hypoxic conditions. These techniques lack reliability and consistency due to a lack of control and visualization. In this research, we developed a smart portable hypoxia chamber that could accurately control the oxygen inside the portable chamber and have a global visualization. The proposed hypoxia chamber provided ease of use with the ranges of 1% to 20% oxygen with increments of 0.5%, as well as reproducibility and accuracy. The chamber displayed great precision on reaching the set oxygen limit and a high stability in maintaining that set level of oxygen compared to the uncontrolled setup for extended durations (24 h). For instance, at a 2% oxygen level, our automated system maintained this level over 1400 min, whereas the oxygen level fluctuated up to 4.5% in the conventional hypoxic chamber. We have also demonstrated the pitfalls of uncontrolled and non-visualized hypoxia chamber setup and the dire need for our system. The hypoxia-induced factor (HIF-1α) expression in cancer cell lines was tested and compared between the conventional hypoxia setup and our automated hypoxia chamber. We observed that there was a twofold increase in HIF-1α expression in the automated controlled chamber compared to the conventional device. The device also provided real-time sensing, visualization and control of the chamber conditions, which could aid in complex in vitro studies.

## 1. Introduction

All cells require oxygen, which plays a crucial role in their survival and functioning. Typically, healthy cells function at a range of approximately 18–21% oxygen in normal physiological conditions [1,2,3], whereas under unusual or diseased conditions, the oxygen percentage significantly depletes around 1–5%, and most tumor or diseased cells tend to survive in a low-to-no oxygen environment. This lack of adequate oxygen is also called hypoxia [4,5,6,7]. Hypoxia and hypoxic signaling are typical characteristics in many diseases, including cancer [8,9], cardiovascular diseases [10,11] and lung disorders [12]. Here, in the case of tumor microenvironments, hypoxia can lead to promoted angiogenesis in tumor tissues, inflammation, migration and metastasis and, finally, treatment resistance [1,4]. A study conducted on the treatment of human breast cancer with radiation therapy concluded that hypoxia was mainly responsible for the resistance of these tumors against radiation therapy. Hypoxia also posed as a challenge in the treatment of certain chemotherapeutic agents due to their dependence on oxygen in order to be cytotoxic [4]. Additionally, researchers have also shown that hypoxic conditions are not just a characteristic, but can also be an essential target for the treatment of these diseases [13]. The hypoxia-induced factor (HIF) is a DNA binding protein complex with a helix–loop–helix structure with a hypoxia-inducible subunit HIF-1α [14]. HIF-1α has been the interest of various researchers due to its correlation with the disease hypoxia. The level of HIF-1α in the microenvironment is a direct indication of the disease progression [15,16,17]. Though various studies have shown the importance of hypoxia in disease progression, replicating the conditions in vitro has always been a hurdle. Researchers have also shown that there is high variation in the HIF-1α level with minimal fluctuations in oxygen level [18].

A challenge thus arises in replicating and maintaining the hypoxic conditions when testing drugs for these diseases, which now becomes a vital component in cancer models and treatment studies [19]. To induce such conditions, hypoxic chambers are used as an alternative to chemical mimetic agents [20,21]. These are the devices that help maintain a specified amount of oxygen condition without compromising cell viability. Hypoxia chambers are fundamental devices in which the cells of interest are placed in an airtight container. The oxygen level is lowered by purging the chamber with hypoxic gas (95% nitrogen and 5% CO_2_). The device would then have to maintain the desired hypoxic environment throughout the course of the experiment without leakages or any fluctuations. The issue with these devices, however, is that there is no inbuilt method to measure or monitor the oxygen levels inside the chamber during the entirety of the experiment, and a need for cost effective and controlled hypoxia chambers then arises. Various research has been conducted to measure and record the oxygen percentage in the chamber during the experiment duration [20,22,23,24]. Though this helps in monitoring the conditions of experiment, it still lacks an aspect of control. We have, therefore, proposed a smart hypoxia device that could not only accurately measure the oxygen composition inside the chamber at any given time, but also automatically pump required gas to maintain the chamber at the set oxygen percentage without need for any additional human intervention. In addition to the above-mentioned advantages, our device also has a very simple effortless design which provides user-friendly operability and portability with a low cost of production.

Research labs commonly use portable hypoxic chambers that are purged with premixed gas composition of 94% nitrogen, 1% oxygen and 5% carbon dioxide (CO_2_) [25,26]. This gas is purged into the chamber for a pre-set duration and sealed until the experiment is completed (approximately 24 to 48 h) with usually no sensor system to monitor the accuracy, which creates uncertainty during experiments. Also, some evaluations require oxygen set at different levels from 1 to 5% depending on the hypoxia requirements [27,28,29]. We have, in this paper, attempted to address these challenges by designing an optimal setup which not only monitors the O2 level, but also maintains the preset oxygen level for the entire duration of the experiment with an additional remote monitoring of the oxygen levels, to provide an uninterrupted and efficient hypoxia control in comparison with the conventional uncontrolled manual hypoxia chamber. Our device’s ability to efficiently maintain the oxygen levels at 1–20% depends on user needs; in this research, 1–5% oxygen is focused due to its relevance in a tumor microenvironment [2].

Studies performed by our automated hypoxic chamber showed that we can set and maintain the level of oxygen inside the chamber at a 1–5% range, with an accuracy of 0.1–0.2% error rate and with a higher rate of precision. With this device, we are also able to monitor the oxygen level throughout the experiment remotely, providing the aspect of reliability. Our setup does not have variability with the number of plates placed inside as the purge is not timed but rather based on a continuous feedback mechanism. Our device would thus function as a cost-effective alternative costing ~$800 compared to high-end hypoxia chambers while still providing higher functionality, portability, operability, remote monitoring [30], automation [31] and data visualization [32].

## 2. Design and Structure

### 2.1. Materials and Components

We have two main hardware units: A sensing board and a control board. In the sensing unit, we use Raspberry Pi 3B+ (Raspberry Pi Foundation) as the computing component. This component is responsible for obtaining data from the oxygen sensor, LuminOx O2 Sensor (SST Sensing Ltd., Coatbridge, UK), and also acts as a web server. We use built-in Wi-Fi to stream data to a PC or smartphone and built-in Bluetooth to send the control signal to the control board. To interact with users, we designed a keyboard (buttons) and LCD screen (I2C LCD1602, SunFounder, Shenzhen, China) as the user interface. Therefore, the user can set up the oxygen level and start monitoring and controlling it using these buttons and LCD screens. The heart of the control board is an Arduino Uno (Arduino Ltd.) attached to the motor driver to direct two valves of oxygen and CO_2_. The Bluetooth module HC-06 (DSD Tech) is interfaced with Arduino Uno via the UART protocol to receive the control signal from the sensing board. Finally, the oxygen sensor is placed inside the hypoxic chamber (Billups-Rothenberg Inc., San Diego, CA, USA) to monitor the oxygen level inside this chamber.

### 2.2. Construction

**Figure 1:**
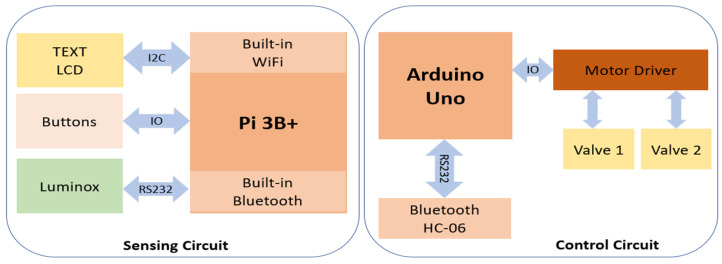
Hardware architecture of the automated hypoxia chamber.

Our system design includes three main components: sensing, control and cloud stream components (Figure 1). We separated the sensing component from the control component since the sensing unit is put inside the chamber, while gas tanks are placed in a different place. By streaming data, users can monitor the oxygen level remotely while processes of sensing and controlling occur simultaneously. The most vital component in this setup is the sensing component. This component contains an O_2_ sensor module that is used to accurately sense the O_2_ level in the chamber. This module is connected to Raspberry Pi 3B+ via a serial port (RS232). The control component consists of Arduino Uno, which bridges the two motors via IO ports. Therefore, Arduino Uno can turn on/off two valves controlled by these two motors. The last component is cloud stream, a bundle of software implemented on Raspberry Pi 3B+ that allows other devices, such as phones, laptops, and personal computers to view past and present data in real time.

After the Raspberry Pi 3B+ reads out the sensor data from the oxygen sensor, it will analyze the value, convert it into control signals and then send it to the control component wirelessly. Specifically, it calculates the error between the expected and current values, then combines the error with the parameters of PID to produce the next command signal. When the control component receives the control signal from the sensing component, it will act accordingly to the control signal. In this case, it closes or opens the valves within an appropriate period. The Raspberry Pi 3B+ collects, stores, and streams data via the internet. It contains a web server written in Python based on the dash framework. Users can track oxygen levels in the chamber remotely by phone or other devices that support a web browser like Google Chrome or Internet Explorer. Users can also view logging data by date, hour and minute to gain an insight into collected data.

## 3. Methods

### 3.1. Experimental Setup

The automated hypoxia chamber is designed with its main characteristics being automation of manual components, system portability, uninterrupted real-time data collection and remote accessibility to system visualization and monitoring. This automated chamber can be set up with ease due to its portable nature and can therefore be installed in a small-to-big laboratory setting.

**Figure 2:**
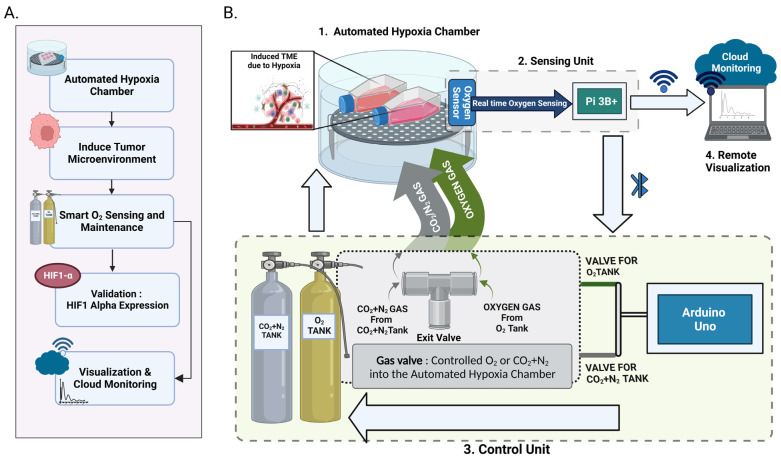
Experimental setup: (**A**) Action flow of the automated hypoxic chamber, (**B**) 1. hypoxia chamber with sensor attached, 2. Sensing unit and LCD Display which is the central operating unit (Pi 3B+) that interconnects the hypoxia chamber, oxygen sensor, control unit and remote visualization unit, 3. Control unit with the microcontroller (Arduino Uno), valves and gas components to purge O_2_ or CO_2_ + NO_2_ into the automated hypoxic chamber with respect to set oxygen levels 4. Remote visualization and monitoring unit (Created with BioRender.com).

Firstly, the unit consisting of the hypoxia chamber and sensor is designed by fitting the sensor into the chamber by carefully drilling a hole and ensuring sealing with silicon as a sealant. The sensor is then placed closer to the gas inlets to make sure precise gaseous flows into the chamber as required. The cell culture flasks will be placed inside the chamber for in vitro experiments. This setup is then placed inside a cell culture incubator. This unit is connected to a gas inlet and outlet, which is in turn, connected to the gas tanks (Figure 2). Two gas tanks are required for the automated operation of the device; the hypoxic gas tank (95% N_2_ and 5% CO_2_) to bring the oxygen level down to the set level and an O_2_ gas tank to set the level if the level drops below the set oxygen level. Once the device is set up, the user can set a varied oxygen percentage ranging from 1% to 21% with an increment of 0.5%. Once the system ensures all components are connected, the Luminox sensor begins collecting the real-time oxygen data [33] and communicates with the sensing unit and the LCD display parallelly displays the current oxygen percentage for the users to make observations. The real-time data collected are simultaneously stored in a .csv file to facilitate data collection and for the user to later analyze the collected data. 

Alongside data storage, the sensing unit continuously communicates with the control unit over Bluetooth to ensure accurate valve function and control the amount of oxygen within the chamber. According to the sensing data received from the oxygen sensor, the valve controls the gas purged, i.e., when the oxygen level in the chamber is higher than the set level, hypoxic gas is purged until it reaches the set oxygen level with a 0.1 error rate. Similarly, if the oxygen level in the chamber is lower than the set oxygen level, then the oxygen gas is purged into the chamber until it is within the threshold range.

An efficient remote data visualization is provided for the real-time observation of data, which the user can access over Wi-Fi in parallel with the system data collection, to provide remote access to the user to monitor the proper functioning of the system and ensure the uninterrupted functioning of the system. If the system performance is interrupted, an alert will then be displayed to the user.

### 3.2. Control System Modeling

The user sets up the reference signal or desired signal. The setup needs to be carried out before starting the system. Otherwise, the default reference signal is used for further control. The reference signal is measured in a percentage of oxygen, and the response signal is also measured in a percentage of oxygen level. The design ensures the system can archive the user’s expectations or avoid extended coverage if the user forgets to set the reference value. The control circuit opens two valves to let an amount of oxygen come into the chamber. For this reason, we need to form a relationship between the period of opening the valves with the added oxygen level in the chamber. Therefore, we need to consider several parameters for each tank: chamber’s volume (v), valves’ size (s_i_), and pressure in the tank (oxygen supply) (p_i_) where i is the valve number (1 or 2). The control signal for each tank is a period of the opening valve where: y_i_ = f (v, s_i_, p_i_, Δt_i_), y_i_ is the oxygen level after opening one valve. For most cases, the chamber’s volume and valves’ size in the tank are not changed due to the form factors. Therefore, our model becomes simpler: y_i_ = f(p_i_, Δt_i_). When we use two valves simultaneously: y = y_1_ − y_2_, where y_1_ is the control function for the oxygen tank, and y_2_ is the control function for carbon dioxide. This formula allows us to model the relationship between the controlling time, pressure and level of oxygen inside the chamber. When the desired oxygen level is hugely different from the current oxygen level inside the chamber, y1 should pump faster with longer periods of time with P gain parameter. However, this may lead to an over-shot issue in which the oxygen level may exceed the desired level (positive error). In this case, we need to use the control function y_2_ to reduce the oxygen level, which may decrease oxygen to the desired level (negative error). If this process is repeated over time, the system finds itself hard to converge. Therefore, we introduce the derivative component as a brake to avoid overshooting. The derivative component helps the system quickly converge, but it may introduce a steady error state, which means the system stops working while the desired oxygen level has not been obtained. Finally, we introduce the integral component to eliminate the steady error state. During experiments, we observed that the system gives the most stability if the pressures in both tanks are similar. Hence, we regulate the pressures in tanks as closely as possible.

### 3.3. Experimental Methodology

Our smart hypoxia chamber was evaluated against a conventional hypoxia chamber to assess the system functionality and its ability to perform real-time sensing of data [34], accurately measuring the oxygen percentage and accordingly controlling the oxygen percentage to set conditions to maintain hypoxia.

We tested the system’s real-time effectiveness by its capability to perform under a set oxygen level in a continuous environment. The system was then subjected to varying oxygen levels to observe the system variability and its effectiveness to adapt to the changes made in a real-time environment, by setting the oxygen levels from low to high (0% to 5%) and vice versa under equal and unequal time intervals and observing the consistency of the system and how it maintains the set oxygen levels. We then determined the accuracy of the system by introducing cells into the chamber and observing the hypoxic behavior of the cells to then determine the effectiveness of our automated hypoxia chamber.

### 3.4. Visualization and Remote Monitoring Working Principle

**Figure 3:**
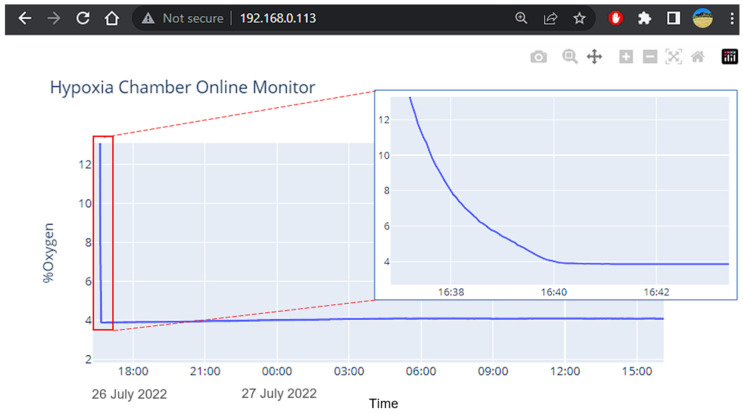
Remote visualization of the data acquired through the server allows user to remotely monitor the current device status during an experiment.

The system offers data visualization and the monitoring of oxygen levels in real-time by a web server running on the sensing control circuit. Since the sensing circuit acquires raw sensor data and processes it, we can either store or stream it over the internet. The technology generates a server chart and converts it into static files, vector images, and text that a web browser can download, interpret, and display correctly. Whenever data are acquired on the server side, the system will notify the client web browser to ensure that data is synchronized between the web server and the web browser (Figure 3). The dash framework provides a server, chart generator, and synchronization mechanisms between the client and server. When a user enters the IP address of the sensing board (server), the application is routed to available data on the server buffer. Then, the chart will be generated in vector image format, which is embedded within a standard HTML (HyperText Markup Language) document. A typical web browser will receive a text document and display it accordingly. On the web browser, the user can select any area on the chart to zoom in/out, pan and download it as typical images.

### 3.5. Precision Test

The precision of the device for the set oxygen level was tested by adjusting the settings to different levels of oxygen percentage (1% to 5%) and measuring the time taken to reach the desired oxygen percentage and if it is maintaining accuracy in doing so. The initial oxygen percentage was brought to room oxygen level (~19–20% oxygen). We then set the oxygen percentages, i.e., 1%, 2%, 3%, 4% and 5%, respectively, with the help of our user-friendly system interface, in separate runs to observe the time the device takes to reach a stable percentage as per the set levels. The sensing unit continuously measured the real-time oxygen percentages within the smart chamber and recorded the data on the device.

### 3.6. Stability and Reliability

The stability and reliability of the smart hypoxia chamber were analyzed here by adjusting different levels of oxygen for a long-term study, and we tested the validity of our device for 24 h timepoint. Our smart automated hypoxic chamber was compared with the uncontrolled conventional hypoxia chamber where 24 h continuous use was monitored, and real-time observations were made to maintain a constant oxygen environment for each oxygen percentage set, i.e., 1%, 2%, 3%, 4% and 5%, respectively, in separate 24 h runs. The stability of the device to maintain the oxygen levels at an extended 48 h timepoint was also tested at each oxygen percentage set, i.e., 1%, 2%, 3%, 4% and 5%, respectively.

### 3.7. Usability Testing for Improved User–End Interactions

Here, we considered the challenge of user–end applicability and make the effortless utilization of our device to minimize frequent human interventions. We tested the usability every time user interruptions were made at randomized time intervals by changing the set oxygen percentage from 1%, 2%, 3%, 4% and 5%, respectively and to observe the system working when the setting is changed from 5%, 4%, 3%, 2% and 1%, respectively, from a higher oxygen concentration to a lower oxygen concentration.

### 3.8. Cell Culture

Two human cancer cell lines A549 (lung adenocarcinoma) and DM-6 (Human Melanoma) from the American Type Culture Collection (ATCC) were used to extract the hypoxia-responsive gene HIF-1α. The cells were cultured at 37 °C using a humidified 5% CO_2_ incubator in Dulbecco’s modified Eagle’s medium (DMEM) with a 10% Fetal Bovine Serum (FBS) for the A549 cells and Roswell Park Memorial Institute (RPMI) with 10% Fetal Bovine Serum (FBS) for the DM-6. The cells were cultured in a T-25 flask until confluency.

### 3.9. HIF-1α Expression Using RT-qPCR Analysis

The cultured cells in the T-25 flasks were placed inside the hypoxia chamber. Two experiments were performed with both automated controlled and uncontrolled conditions for 24 h and 48 h under low serum media [28]. The total RNA was isolated from cells using Monarch^®^ Total RNA Miniprep Kit. cDNA was synthesized from total RNA using iScript™ Reverse Transcription Supermix for RT-qPCR (BioRad, Hercules, CA, USA) according to the manufacturer’s instructions. The primer sequences for the specified genes [35] are shown in Table 1. The RT-qPCR reaction was performed using iTaq™ Universal SYBR^®^ Green Supermix (Biorad) according to the manufacturer’s protocol. The data were normalized to cells cultured in Normoxia.

## 4. Results and Discussion

### 4.1. Device Precision to Reach a Set Oxygen Level

**Figure 4:**
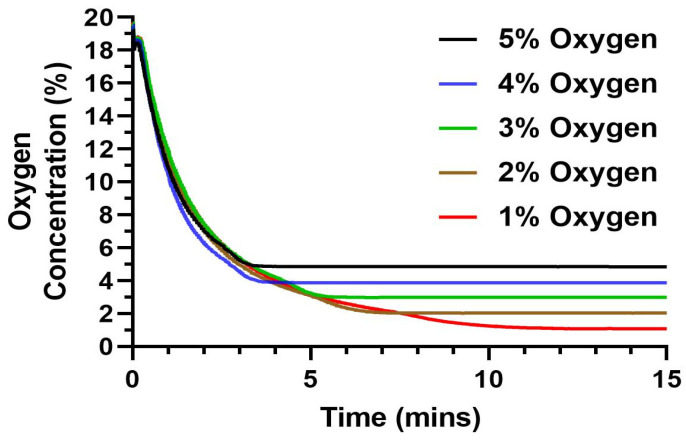
Precision testing of the automated hypoxic chamber at different set oxygen levels from 1–5% where the device lowered and maintained the microenvironment to the set oxygen level with an error of <0.1%.

Testing treatment against the disease or testing the mechanism of the disease itself requires accurate replication of the disease microenvironment in vitro. In the case of hypoxia-dependent diseases, even a small difference in the oxygen percentage might change the cellular activity and function [36]. Thus, we analyzed the capability of the automated hypoxic chamber in reaching the set oxygen percentage required for the experiments. We tested a range of 1–5% with an increment of 1% oxygen level due to its relevance in a tumor microenvironment (Figure 4) [2]. We observed that the device was highly precise in lowering the chamber oxygen level to the required set levels for the tested range of 1–5%. The set levels were reached in less than 5 min for the 4 and 5% oxygen, with a gradual increase in time as we moved towards the lower oxygen percentage. We observed that to reach a 5% oxygen level, the device took ~3 min with a gradual increase in time as we set lower oxygen levels. The device took ~9 min to reach the lowest set oxygen level (1%). Thus, the device will operate in a time range of <10 min for any set level of oxygen.

The precision of the device was further tested by analyzing how much deviation the measured value was from the set value. We observed from multiple repetitive runs that the device had a precision error ranging from 0.04–0.14, with an average error < 0.07% oxygen. Thus, we can validate the capability of the device to both reach the set oxygen percentage with high precision and in a very short time.

### 4.2. Stability and Reliability

**Figure 5:**
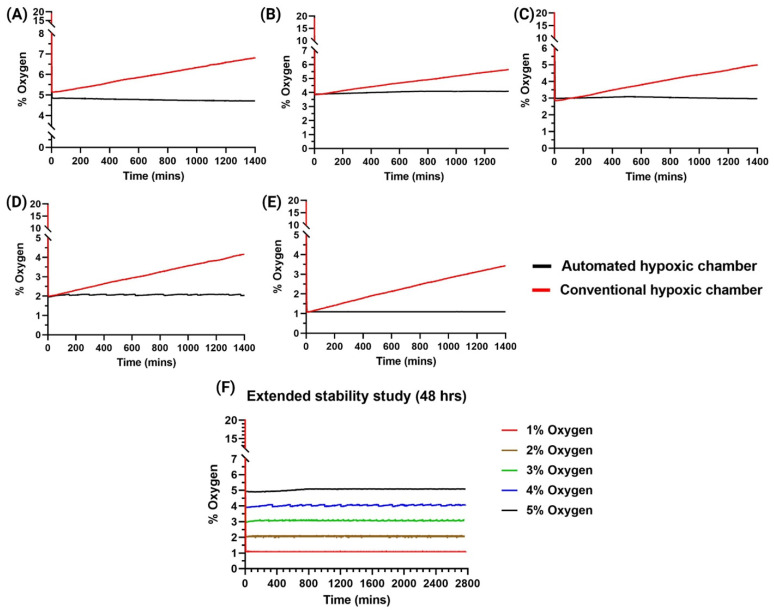
Stability and reliability studies at different set oxygen levels. (**A**) 5%, (**B**) 4%, (**C**) 3%, (**D**) 2%, (**E**) 1% where the automated hypoxic chamber showed reliable stability for 24 h compared to that of the conventional uncontrolled hypoxic chamber. (**F**) Extended stability study for 48 h with the automated hypoxic chamber at different oxygen levels showed reliable stability in oxygen control.

Many studies surrounding in vitro therapeutics depend on the extended analysis of the cells/tissues. Most of these experiments including proliferation, cytotoxicity studies extend to at least 24 h. We, thus, wanted to analyze if the automated hypoxic chamber was able to maintain the set oxygen levels accurately for the extended 24 h timepoint. We observed that for any set oxygen percentage, the non-controlled hypoxic chamber did not maintain the set level with a single purge (Figure 5). As shown in the figure, the unregulated chambers had a gradual increase in their oxygen levels with some reaching almost 3% more than that of the set level. This is a very common phenomenon observed in conventional hypoxic chambers, which could be due to various factors including cellular interactions, temperature-dependent concentration change or even leakage to the external surroundings [37]. This has been a common observation in various literature where multiple gas purges are required for maintaining the set oxygen percentage [26]. This is very inconvenient for the user and most often does not produce reliable and repeatable data. There is also a need for extensive optimizations necessary for consecutive purging. These graphs also clearly indicate that there is a certain need for the user to be present at all times, monitoring the oxygen % of the chamber and purge gas at various intervals of time.

On the other hand, we can observe that with our automated hypoxic chamber, the oxygen levels were maintained with high stability. For extended time points, we can observe that the oxygen levels were maintained with an error of <0.1% oxygen. We also observed that there were multiple purges required to maintain the set oxygen level with an average time of 90 min between each purge. We also performed an extended stability analysis at various oxygen levels to show the ability of the automated hypoxic chamber to maintain the oxygen levels at the set level for an extended duration of 48 h (Figure 5). We observed that for all oxygen levels, the oxygen level inside the device was very stable with an error of <0.1% oxygen. These data clearly show that our device not only solves the issues associated with extended hypoxia studies, but also makes it completely automated, eliminating the manual work and errors associated with it.

### 4.3. Usability Testing for Improved User–End Interactions

Some experiments might require different oxygen levels at different timepoints, and user interventions in the middle of experiments should not interfere with the functionality. In this experiment, we have tested the usability of the device when modifying the oxygen levels mid-experiments. We performed a step-up and a step-down interaction.

**Figure 6:**
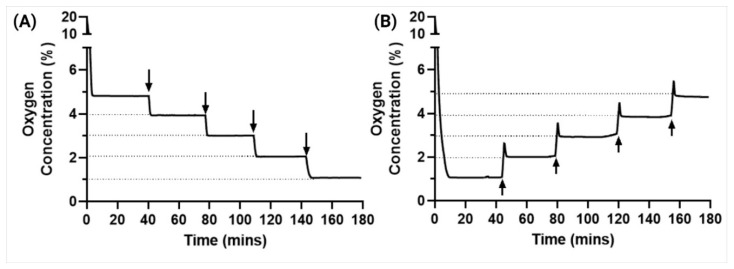
Device usability testing by changing the oxygen levels mid experiment by (**A**) step-down transition and (**B**) step-up transition shows the ease of operability for the user.

For experiments that require the user to have different levels of oxygen, we also tested the device’s capability to transition to a set level of oxygen from a pre-set level of oxygen. We observed that the device took less than 5 min to reach the new set level with very high precision of less than 0.1% error and was also able to maintain the new oxygen level (Figure 6). We did observe that when going from lower levels to higher levels of oxygen, there was a slight increase in time for the device to become stable compared to that of going from higher to lower oxygen levels; however, this difference was not significant and hence, no further analysis was performed. This can, however, be avoided by reducing the inflow rate of the oxygen gas. Thus, we showed the ease of the system’s variability and reliability on the user–end and increased operability of our smart hypoxia chamber.

### 4.4. In Vitro Evaluations by HIF-1α Expression Using RT-qPCR Analysis

**Figure 7:**
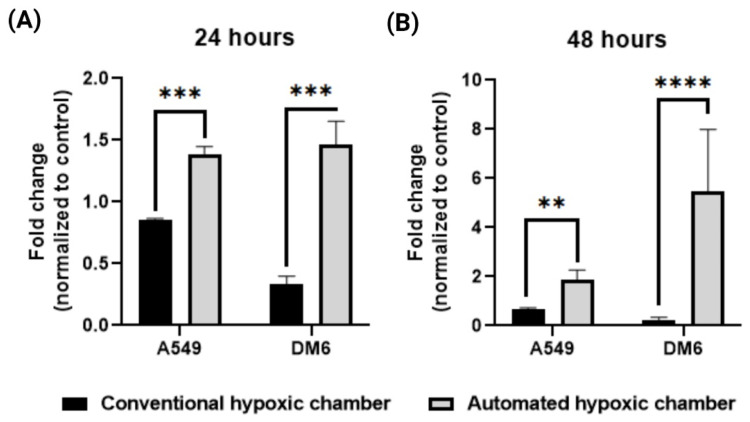
RT-qPCR analysis of cancer cell lines (A549, DM6) treated for (**A**) 24 h, (**B**) 48 h in the automated hypoxic chamber shows significantly higher expression of HIF-1α expression compared to that of cells treated in the conventional hypoxic chamber (** *p* < 0.01, *** *p* < 0.001, **** *p* < 0.0001).

Though all the testing mentioned above showed enhanced functionality and precision of the device, testing the real-life application of the device becomes absolutely necessary [22]. We tested the device’s capability in vitro to effectively create a hypoxic condition to cells cultured within the chamber and compared it to that of a non-controlled device. It is known that a hypoxic environment induces the overexpression of HIF-1α [38] and the level of HIF-1α is in direct correlation to the hypoxic level [18]. Multiple researchers have shown how the HIF1α expression of these cancer cells corelates with respect to hypoxic conditions [39,40]. To determine the effectiveness of the chamber, we tested two cancer cell lines namely A549 and DM6 in vitro for their gene expression of HIF-1α after 24 h of treatment in the hypoxic chamber (Figure 7). We demonstrated that there was a significant increase in the HIF-1α expression for both the cell lines when compared to that of the uncontrolled hypoxic chamber. As shown in Figure 7, the HIF-1α expression of DM6 was significantly lower than that of A549 when treated in a conventional hypoxic chamber. On the other hand, we observed that the true HIF-1α expression of both cells WAS similar and significantly higher when maintained in the automated hypoxic chamber. In addition, the HIF-1α expression was more than double for that of A549, where the conventional chamber had an 0.8-fold increase compared to normoxic condition, cells treated in the automated hypoxic chamber showed around 1.4-fold increase in HIF-1α expression, this trend was more significantly observed in the DM6 cell line where the expression levels of conventional system treated cells were 0.3-fold increase from normoxia, cells treated in our automated hypoxic chamber showed 1.5-fold increase in HIF-1α expression. This effect was even more pronounced in the 48 h study where A549 cells in the automated hypoxic chamber produced a 1.9-fold increase in HIF-1α expression compared to 0.6-fold in conventional system. Interestingly, we observed a much more profound effect in the DM6 cells after 48 h where cells in the conventional system showed a 0.3-fold increase in HIF-1α expression, and cells in the automated hypoxic chamber displayed a 5.4-fold increase in HIF-1α expression. These expressions were normalized to that of normoxia levels, and we can see that the automated hypoxic chamber can produce a more effective microenvironment for the cells compared to that of a single purged uncontrolled system.

## 5. Conclusions

In this paper, we have presented our innovative platform for developing a smart portable hypoxic chamber with accurate sensing, control and visualization of in vitro cell culture for replication of a cancer microenvironment. Our novel chamber is equipped to effectively induce the tumor microenvironment conditions in vitro, aiding therapeutic studies that can bridge the gap between laboratory and clinical studies. Where maintaining the hypoxic condition poses a challenge during in vitro studies for these diseases and becomes essential by targeting hypoxia for the treatment of diseases, which our system can achieve effortlessly with a user-friendly approach. Lastly, our device not only accurately measures the oxygen level within the chamber in real-time, but also automatically pumps gas as required to maintain the chamber at the set oxygen percentage stably while having the ability to remotely monitor the device over the cloud for both short- and long-term studies providing great reliability for scientists to conduct hypoxia experiments with ease. The system will be further upgraded with the addition of the CO_2_ sensor and triple valve connections to accurately measure and maintain both O_2_ and CO_2_ gases simultaneously for tailored replication of the disease environment.

## Figures and Tables

**Table 1:** Primer sequences for RT-qPCR [35].

Gene		Sequence
**HIF-1α**	Forward	(5′-CCTGCACTGAATCAAGAGGTGC-3′)
	Reverse	(5′-CCATCAGAAGGACTTGCTGGCT-3′)
**GAPDH**	Forward	(5′-TCCTCCTGTTTCATCCAAGC-3′)
	Reverse	(5′-TAGTAGCCGGGCCCTACTTT-3′)

## Data Availability

The data presented in this study are available on request from the corresponding author. The data are not publicly available due to intellectual property related concerns.
